# Rikkunshito and Ghrelin

**DOI:** 10.1155/2010/283549

**Published:** 2010-01-26

**Authors:** Tomohisa Hattori

**Affiliations:** Tsumura & Co., Tsumura Research Laboratories, 3586 Yoshiwara, Ami-machi Inashiki-gun, 300-1192 Ibaraki, Japan

## Abstract

Rikkunshito is a popular Japanese traditional medicine that is prescribed in Japan to treat various gastrointestinal tract disorders. In a double-blind controlled study, rikkunshito significantly ameliorated dysmotility-like dyspepsia and brought about a generalized improvement in upper gastric symptoms such as nausea and anorexia when compared with a control group. Several studies in rats have shown enhanced gastric emptying and a protective effect on gastric mucosa injury with rikkunshito administration. In addition, rikkunshito in combination with an anti-emetic drug is effective against anorexia and vomiting that occur as adverse reactions to chemotherapy in patients with advanced breast cancer. However, the mechanism by which rikkunshito alleviates gastrointestinal disorders induced by anticancer agents remains unclear. It has recently been shown that rikkunshito ameliorates cisplatin-induced anorexia by mediating an increase in the circulating ghrelin concentration. Moreover, Fujitsuka et al. found that decreased contractions of the antrum and duodenum in rats treated with a selective serotonin reuptake inhibitor were reversed by rikkunshito via enhancement of the circulating ghrelin concentration. These findings show that rikkunshito may be useful for treatment of anorexia and may provide a new strategy for improvement of upper gastrointestinal dysfunction.

Rikkunshito is one of the few traditional Japanese medicines for which a double-blind study has been conducted. This trial was performed in patients with dysmotility-like dyspepsia [1, 2] based on a report that rikkunshito showed efficacy against non-ulcer dyspepsia, which is an old diagnostic classification. In a subsequent comparative clinical study, rikkunshito was found to be more effective than cisapride against undefined gastrointestinal complaints such as chronic gastritis [3]. Rikkunshito is prepared by compounding eight herbal medicines listed in the Japanese Pharmacopoeia: *Atractylodis Lanceae Rhizoma*, *Ginseng Radix*, *Pinelliae Tuber*, *Hoelen*, *Zizyphi Fructus*, *Aurantii Nobilis Pericarpium*, *Glycyrrhizae Radix* and *Zingiberis Rhizoma*. It has recently been shown that oral administration of rikkunshito stimulates secretion of the orexigenic peptide, ghrelin, from the stomach [4, 5]. In this section, the effects of rikkunshito are introduced, with main focus on the action of rikkunshito as an enhancer of ghrelin secretion.

Anorexia is commonly seen in gastrointestinal diseases, although it is not a specific symptom. Anorexia is particularly common in chronic gastritis and gastric cancer but also occurs in acute hepatitis, hepatic cirrhosis, chronic pancreatitis, and chronic cholecystitis. Harasawa et al. [1] showed that rikkunshito promotes improvement of anorexia in a double-blind study in patients with functional dyspepsia (FD). A combination of rikkunshito plus serotonion (5-hydroxytriptamine, 5-HT)and 3 receptor antagonist (an anti-emetic agent) reduced anorexia and vomiting induced as adverse reactions after chemotherapy in patients with advanced lung cancer, compared with administration of the anti-emetic agent alone [6]. Similarly, administration of a selective serotonin reuptake inhibitor (SSRI), fluvoxamine, in combination with rikkunshito for eight weeks resulted in a significant reduction in the number of patients who complained of adverse events, especially retching, compared with SSRI administration alone [7]. The gastrointestinal symptom rating score also significantly improved within two weeks of starting coadministration of the SSRI with rikkunshito [7]. These findings suggest that rikkunshito suppresses the onset of adverse reactions to frequently prescribed drugs that cannot be treated adequately by adjuvant therapy with current Western medicines.

Cisplatin has been shown to cause a significant decrease in plasma ghrelin and food intake in rodents [4], and intravenous injection of exogenous acylated ghrelin inhibited the decrease in food intake after cisplatin administration. Rikkunshito also inhibited the decrease in circulating ghrelin concentration and ameliorated the decrease in food intake caused by cisplatin. Interestingly, coadministration of a ghrelin receptor antagonist, [D-Lys^3^]-GHRP-6, with rikkunshito abolished this effect. These findings suggest that the mechanism of improvement of anorexia by rikkunshito may involve ghrelin receptor activation via stimulation of ghrelin secretion from the stomach into the plasma. Heptamethoxyflavone, an active ingredient flavonoid in rikkunshito, has been shown to have a pivotal effect on stimulation of ghrelin secretion. In addition, P388-bearing mice showed a tendency for improved survival with rikkunshito treatment, and survival was further improved by treatment with cisplatin in combination with rikkunshito, although the difference was not significant [8]. These results show that administration of rikkunshito has no adverse effect on the anticancer action of cisplatin itself.

Fujitsuka et al. showed that oral administration of rikkunshito restores disturbed motor activity in the gastrointestinal tract and improves anorexia in rats administered SSRIs [5]. Intraperitoneal administration of fenfluramine or fluvoxamine shifted fasted rats from a fasted-like motor pattern in the antrum and duodenum to fed-like motor activities similar to those seen after feeding. A significant decrease in the plasma concentration of acylated ghrelin, delayed gastric emptying, and decreased food intake were also observed after administration of the SSRI. Concomitant oral administration of rikkunshito with an SSRI suppressed the decrease in plasma acylated ghrelin, changed the fed-like motor activity to fasted activity, improved anorexia, and enhanced gastric emptying. These effects were abolished by coadministration of a ghrelin receptor antagonist with rikkunshito.

Cisplatin and SSRIs are widely used in clinical practice. 5-HT is a key factor in adverse reactions to these drugs, since both stimulate production of excess 5-HT and suppress 5-HT metabolism in vivo. Thus, it is of interest that involvement of the 5-HT2 receptor in appetite control has recently been shown [9]; specifically, appetite is suppressed when the 5-HT2B receptor in gastric smooth muscle and the 5-HT2C receptor in the central nervous system are activated by receptor agonists. 5-HT produced during treatment with cisplatin or SSRIs binds to various receptor subtypes and is likely to stimulate the 5-HT2B and 2C receptors. A decrease in plasma ghrelin is suppressed by administration of antagonists for these receptors, leading to improvements in food intake and gastrointestinal dysmotility [4, 5]. Isoliquiritigenin, heptamethoxyflavone and hesperidin are ingredients of rikkunshito that have been shown to antagonize 5-HT2B and 2C receptors [4]; thus, these ingredients are considered to play an important role in the improvement of appetite by rikkunshito. Administration of hesperidin reverses the decrease in plasma ghrelin in cisplatin-treated rats and shifts the fed-like motor pattern induced by SSRI administration to a fasted pattern. Thus, 5-HT2C antagonism by active components in rikkunshito may lead to the improvement of anorexia.

More recent report demonstrated that administration of rikkunshito improve anorexia of aging via inhibiting a reduced hypothalamic ghrelin receptor reactivity [10]. The data indicated that aging-associated anorexia is caused by an increase in plasma leptin, which results from disturbed reactivity of ghrelin in the hypothalamus and regulation of ghrelin secretion. Oral administration of cilostamide, phosphodiesterase type 3 (PDE3) inhibitor improved anorexia in aged mice. The components of rikkunshito (nobiletin, isoliquiritigenin, and heptamethoxyflavone) had inhibitory effects on PDE3 activity. Dysregulation of ghrelin secretion and ghrelin resistance in the appetite control system occurred in aged mice and that rikkunshito ameliorated aging-anorexia via inhibition of PDE3.

In summary, administration of rikkunshito stimulates secretion of ghrelin from stomach via peripheral 5-HT2B and central 5-HT2C receptor antagonism as seen in Figure 1. Moreover, rikkunshito is also an enhancement of ghrelin receptor reactivity via PDE3 inhibition. These studies raise the possibility of using rikkunshito to treat anorexia.

## Figures and Tables

**Figure 1:**
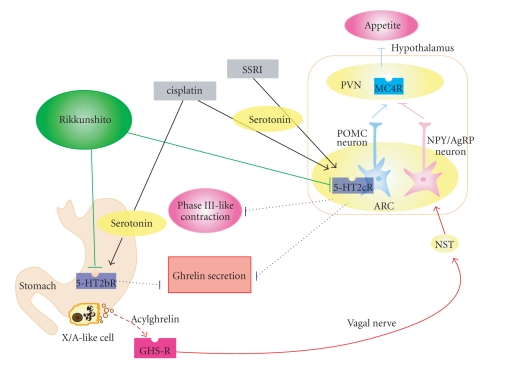
Schematic diagram of action mechanisms of rikkunshito on appetite.
